# Sustained and Universal Fertility Recuperation in Kazakhstan

**DOI:** 10.1007/s10680-023-09671-6

**Published:** 2023-07-13

**Authors:** Maxim Kan

**Affiliations:** Demography Unit, Department of Sociology, Stockholm University, 106 91 Stockholm, Sweden

**Keywords:** Demographic transition, Fertility, Ethnicity, Post-Soviet, Kazakhstan, MICS

## Abstract

The fertility rates of Kazakhstan have reversed to levels not seen for several decades. The striking fertility increase poses questions regarding the extent to which this new development is shared across socio-demographic groups and the nature of fertility recuperation. The current study employs UNICEF Multiple Indicator Cluster Survey data and event-history modelling to analyse parity progressions to one, two, three, and four children. The results suggest a sustained fertility increase that is not merely associated with the recuperation of delayed first births, but a genuine increase across all birth orders. This pattern is evident for both main ethnicities in Kazakhstan and across educational groups. The gradual increase of higher-order births, especially among ethnic Kazakhs, indicates a reversed fertility transition and also that the previous fertility decline in the 1990s was not part of a general transition towards below-replacement fertility but rather a reflection of economic crisis after the collapse of the Soviet Union.

## Introduction

The Total Fertility Rate (TFR) of Kazakhstan has recently been on a roller coaster, dropping from 2.84 in 1989 to 1.80 in 1999 and rebounding back to 3.00 in 2018 (Demoscope, 2019). These strong fertility fluctuations may appear puzzling at first sight but can be linked to several aspects of social change in Kazakhstan during the transition from a Soviet republic to an independent post-socialist state. The decline in fertility in the 1990s can be attributed to the economic crisis and restructuring of society during the transition to a market economy in the 1990s (Billingsley, 2010; Spoorenberg, 2015). Industries disappeared, inflation skyrocketed, and unemployment and wage arrears were widespread (Alam & Banerji, 2000). In addition, many institutional features such as childcare provision (Information-Analytic Centre, 2017), maternity leave, and other forms of social benefits were significantly reduced during the 1990s (Werner et al., 2017). In contrast, economic measures improved substantially in the 2000s, mostly due to increasing oil prices and foreign investments in extraction industries. The TFR appears to have followed the economic growth of the country. Indeed, a study by Spoorenberg (2015) suggested that 81% of the TFR increase from 2000 to 2011 was due to economic growth.

The more recent developments are also noteworthy because aggregate fertility measures during the 2010s show a sustained upward trend. The dramatic recovery in TFR seems to contradict the irreversibility argument of the logic of both the first and the second demographic-transition frameworks (Coleman, 2006; Lesthaeghe, 2020). Recent TFR data indicate that the fertility recuperation studied in Spoorenberg (2015) has persisted over quite an extended period. Whereas Spoorenberg (2013, 2015) relied on aggregate data, this study employs individual-level data on parity progressions that will allow to gain better insight into the underlying patterns of recent fertility change. Individual-level data from records of fertility histories can unravel patterns of fertility change that are not masked by compositional changes in the population at hand and allow to explore the extent to which fertility trends have been universal or specific to different sub-groups of the population.

Specifically, it has been argued that the sweeping changes in Kazakhstan since the collapse of the Soviet Union have affected the two main ethnic groups in the country differently (Agadjanian, 1999; Agadjanian et al., 2008, 2013). Ethnic Russians and ethnic Kazakhs have long had very different fertility profiles, where the former group appears to be on track for a situation that is normally ascribed to a Second Demographic Transition (Lesthaeghe & Surkyn, 2004), similar to co-ethnics in Russia (Zakharov, 2008). Meanwhile, ethnic Kazakhs may still be experiencing fertility change that could belong to patterns of fertility decline that occur during an initial demographic transition. Different reactions to economic and social development may also have induced different fertility reactions for different socioeconomic groups in Kazakhstan. In particular, it is imperative to study how fertility developments and recent fertility increases have been at play for women of different levels of educational attainment.

In summary, this study extends previous research on reproductive behaviour in Kazakhstan by using more recent demographic data that stretch into the 2010s, employing event-history analyses of parity-specific fertility transitions, and analysing educational differentials in fertility change. In the study, the following research questions are addressed: (1) Was the fertility increase that occurred in the early 2000s a temporary deviation from previous trends or part of a behavioural change that reflects a more long-term return to more elevated fertility? (2) Was the pattern of fertility recuperation a universal development or did it belong to specific subgroups of the population, reflecting ethnic and educational differences in behavioural change?

## Literature Review/Theoretical Perspectives

### Demographic Transitions

To understand the fertility changes that were observed in Kazakhstan in the 1990s and 2000s, it is first worth positioning these developments within the frameworks of demographic transition theory. Demographic transition theory assumes a development from high mortality and fertility towards low mortality and fertility (Notestein, 1945). This happens as countries make significant progress in reducing mortality, including among infants (Kirk, 1996). More surviving children thus indirectly influence fertility considerations. Furthermore, with the development of more efficient contraceptive methods, people gain more control over family planning and can better regulate their fertility (Coale, 1984).

Following the path assumed by demographic transition theory, the TFR in Kazakhstan decreased from 4.5 in 1959, which was one of the highest levels among the Soviet Republics, to 2.91 in 1981. However, comparisons of aggregate TFR estimates for Kazakhstan across time points are not very informative because massive shifts in the composition of the population occurred at these times. In particular, the 1960s were associated with a big influx of Russian and other Slavic people from the European parts of the Soviet Union moving to Kazakhstan. It is presumed that these individuals had already completed their fertility transition by that time (Zakharov, 2008). To disentangle Kazakhstan’s fertility trends, more disaggregated data than those based on national averages is needed.

A below-replacement level of fertility in Kazakhstan was achieved in the 1990s. This could have been regarded as evidence of the completion of the country’s demographic transition, which then would have involved a stabilization of fertility around this level. Unexpectedly, however, the TFR increased gradually in the 2000s to levels only seen decades earlier. This development casts doubt on whether the fertility decline in Kazakhstan during the 1990s belonged to the final phases of the country’s demographic transition.

Even though aggregate fertility levels show that the decline in the 1990s was temporary, it is possible that at least some segments of the country's population experienced the final stage of the classical demographic transition (Blue & Espenshade, 2011) or were even part of developments typically associated with the Second Demographic Transition. (Lesthaeghe & Surkyn, 2004). The Second Demographic Transition (SDT) is associated with a substantial decline in period fertility rates due to increasing levels of individualization, shifts in values and attitudes, the postponement of marriage and first childbearing, and increases in nonmarital childbearing (Lesthaeghe & Surkyn, 2004). Already in the mid-twentieth century, Russian and other Slavic people differed from the titular ethnicity of Kazakhstan in terms of familial ties, family size, aggregate fertility, and gender roles. As a result, these groups of people could have served as forerunners in the formation of new values in the country, which were also shared by their co-ethnics in Russia (Zakharov, 2008). Moreover, recent studies show that one-child families have become the most prevalent family form in Russia (Frejka & Gietel-Basten, 2016), and the fertility levels of ethnic Russians are among the lowest in Russia (Kazimov & Zakharov, 2021). Considering the differences in fertility developments between ethnic groups in relation to both the first and the second demographic transitions may thus be relevant in the case of Kazakhstan.

On the other hand, propositions derived from evolutionary theory have raised questions about the irreversibility of both the first and second demographic transitions. Burger and DeLong (2016), for example, argue that demographic behaviour is partially influenced by genetic factors, which cause people to respond to various social and ecological conditions. Since these conditions can change in different ways, fertility levels may increase or decrease instead of remaining stable. Furthermore, Burger and DeLong (2016) contend that changes in cultural norms do not always lead to low fertility. In the case of Kazakhstan, one can observe a combination of new lifestyles influenced by Western societies after the collapse of the Soviet Union, as well as a reassessment of its cultural heritage, traditions, and religion, which could affect childbearing behaviour in different directions.

Some of the proponents of the classical demographic transition theory also highlighted the delaying effect of religion on the onset and speed of fertility change. Thus, Coale (1984) pointed out that the fertility decline in Central Asia happened later than in other parts of the Soviet Union and argued that Muslim culture could be particularly resistant to lower fertility. Similarly, Kirk (1996) argued that Muslim countries had been slower to enter the fertility transition, while more recently also Lesthaeghe (2020) questioned the applicability of the SDT framework to the context of patriarchal Muslim countries. Considering the changes in the ethnic composition of the country and the increase in religiosity and restoration of traditions and customs among ethnic Kazakhs (Aydıngün, 2007; Telebaev, 2003; Yerekesheva, 2020), it can be assumed that these factors also have an impact on fertility developments and demographic transition.

### The Procyclical Relationship of Fertility with Business Cycles

Apart from the demographic transition arguments, economic developments may shed light on fertility change in the country. A procyclical relationship between period fertility measures and the business cycle would mean that the aggregate fertility rate may drop during an economic recession while it can increase during periods of economic growth. This has been found empirically in many developed countries (Andersson, 2000; Karaman Örsal & Goldstein, 2018; Sobotka et al., 2011). A similar procyclical association has also been found in post-communist settings (Kohler & Kohler, 2002; Sobotka, 2011). Furthermore, Perelli-Harris (2005) associated low fertility in Ukraine with persistent stopping behaviour after a first birth in relation to an economic crisis. Billingsley (2010) also found that economic crisis in the post-socialist region was associated with stopping behaviour in childbearing, while economic improvement was associated with birth postponement. In a study on Central Asia and Kazakhstan, Spoorenberg (2015) found that fertility was procyclically associated with the growth of the Gross Domestic Product (GDP) and that the increase in fertility rates was not merely due to a reduced pace of childbearing postponement.

However, one would not expect fertility to continue increasing as long as there is economic growth. Under improved economic conditions, parents may have more resources to support more children. But apart from better affordability of childrearing under improved economic conditions, people also face higher opportunity costs. Thus, it is expected that fertility will recuperate during economic improvement but then stabilize at certain levels: either at replacement-level fertility or at levels where the country is otherwise situated during its course of demographic transition.

### Educational Attainment and Fertility

#### Education Within Demographic Transition Theory

According to classical demographic transition theory, increased education among women is associated with fertility decline through the postponement of marriage and first births (Kirk, 1996). In addition, it has been suggested that the timing of fertility decline during the demographic transition is influenced more by women's education than purely by economic factors of modernization. The transition is also closely associated with the diffusion of new ideas rather than solely economic development (ibid.). Simultaneously, increases in the returns to education result in higher spending on education, making childrearing more expensive. As a result, parents tend to allocate more resources to each child, leading to a decrease in the number of children (Becker, 1981).

#### Education and Economic Cycles

The reaction to an economic recession or to economic growth, and how it is associated with fertility, may also depend on women’s and men’s educational attainment. More educated women are more likely to postpone childbearing during times of economic recession to avoid a decrease in income and maintain career stability (Becker, 1981; Sobotka et al., 2011). In contrast, less educated women may find it even more difficult to get employment during an economic recession and could strive for another ‘strategy’ such as childbearing, especially if it is accompanied by some state financial support (Friedman et al., 1994; Sobotka et al., 2011). Thus, an economic recession may stimulate fertility increase among the less educated and fertility decrease among more educated women. Similarly, Kreyenfeld (2016) found that a secure economic situation is not a uniform prerequisite for childbearing and that this is more important for educated women as well as those who start their families at later ages. Comolli et al. (2021) instead showed a reversal from heterogeneity to homogeneity in educational differences in birth hazards in relation to the economic uncertainty of the Great Recession of 2008–2009 in the Nordic Region.

In the post-Soviet context, Billingsley (2011) found a uniform decline of second birth rates in Russia within educational groups and occupational classes during the economic crisis, and that the rates did not increase to pretransition levels during the early years of economic recovery (up to 2004). Considering the above-mentioned empirical findings and the transition to a market economy in Kazakhstan during the turbulent 1990s and the subsequent economic improvement during the 2000s, one can assume that people with different educational levels could display different fertility patterns and trends of fertility change.

In the next section, I present the background in terms of changes in the economic context in Kazakhstan from 1991 to 2015, as well as changes in family policies and the ethnic composition in the country during the period. This forms the basis for the current study, situating the aforementioned theories and empirical findings within the context of Kazakhstan.

## The Case of Kazakhstan

### Economy

**Fig. 1:**
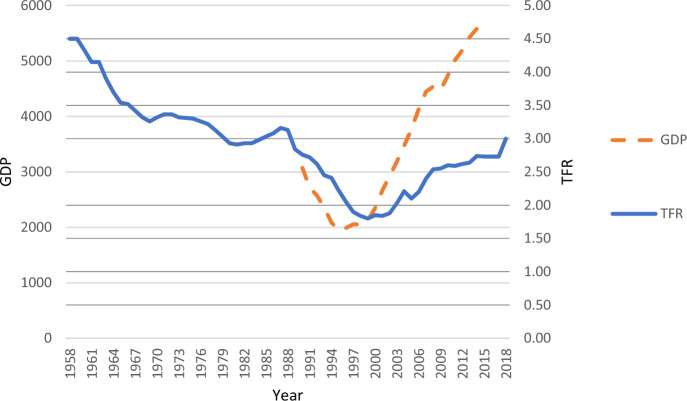
GDP per capita, 1990–2014 and TFR in Kazakhstan, 1958–2018. *Source* TFR constructed using data from Demoscope (2019) and TransMonee Database (2020)

Figure 1 shows that changes in the TFR have been following changes in the GDP of Kazakhstan. Since the collapse of the Soviet Union, and during the transition to a market economy, the TFR steadily declined to an under-replacement level for most of the 1990s. After the turn of the millennium, the TFR rebounded to levels last seen in the late 1980s.

Gaining independence and transitioning to a market economy was first associated with a significant economic crisis. The entire economy, which was centrally planned during the Soviet period, required restructuring, leading to the disappearance of numerous industries and jobs. As a result, returns to education became insignificant for many people. The turbulent 1990s in Kazakhstan were marked by hyperinflation and a high level of unemployment. Even those who were employed either experienced months-long salary delays (wage arrears) or received non-cash contributions as their salaries. (Alam & Banerji, 2000). Thus, the standards of living deteriorated significantly, income levels dropped, and GDP per capita decreased by at least a third during the 1990s compared to the pre-independence era. However, Kazakhstan later made substantial progress, achieving economic growth that enabled the country to surpass the GDP levels of the late Soviet Union in the early 2000s and nearly double its GDP per capita by 2014 (TransMonee Database, 2020).

### Family Policies

Early independence years were also associated with a deterioration of the social protection that people were used to during Soviet times. As a result, the number of preschools was drastically reduced. The number of preschool settings in Kazakhstan was eight times lower in 2000 than in the year preceding the collapse of the Soviet Union (Information-Analytic Centre, 2017). Kazakhstan was only able to match the number of preschool settings of 1990 in 2015. The participation rates for 3–6-year-olds in early childhood education and care were above 50% during the years before independence and dropped dramatically, to 12%, in most of the turbulent 1990s (TransMonee Database, 2020). Only from the early 2000s has there been a gradual increase in enrolment rates, and the pre-independence rates were only achieved in the early 2010s. The availability of childcare services can have a significant impact on decisions regarding fertility, as demonstrated in studies conducted in various countries such as Spain (Baizan, 2009), Norway (Rindfuss et al., 2010), Japan (Fukai, 2017), and Belgium (Wood, 2019; Wood & Neels, 2019). Therefore, it is reasonable to assume that childcare provision could also influence fertility decisions in Kazakhstan.

There have been several changes in policies related to childbearing during the time analysed in this study. Initially, in 1981, the Soviet Union implemented a pronatalist policy which introduced a financial allowance specifically designed for first-time mothers [for a more comprehensive understanding of this policy, refer to Zakharov (2008)]. Due to the fact that payments to pregnant women and mothers were disbursed by employers, there was a growing likelihood during the 1990s that they would experience delays in receiving their payments, given the widespread issue of wage arrears. In 1999, a new Labour Law was enacted, which reduced the duration of unpaid leave and limited job protection to 18 months from the previous 3 years. However, in 2007, a new Labour Code was introduced, reinstating the provision of 3 years of unpaid leave. Moreover, the State Social Insurance Fund provided a new allowance equivalent to 40% of an individual's pre-birth income, which was granted for a period of 12 months following childbirth (Legal information system of RK, 2022). Consequently, these legislative revisions have the potential to influence decisions regarding fertility, and their implementation is closely associated with the economic fluctuations experienced in the country.

### Ethnic Composition

**Fig. 2:**
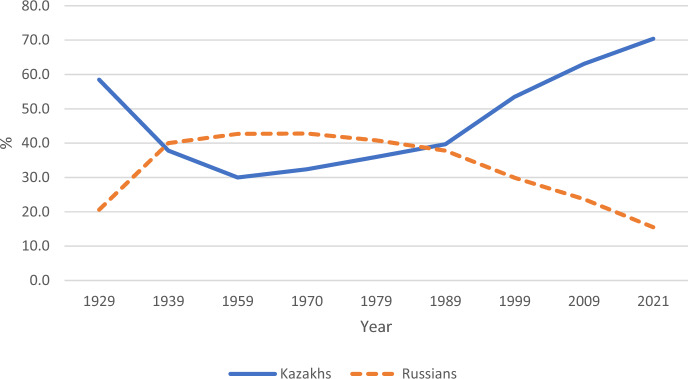
Population composition of Kazakhstan, 1929–2021, %. *Source* Constructed using data from Agency of Statistics of the Republic of Kazakhstan (censuses 2009, 2021) and Demoscope Weekly USSR historical data

The ethnic composition of Kazakhstan should also be considered when studying fertility trends in the country. Several studies (Spoorenberg, 2013, 2015) point out that changes in the ethnic composition of Kazakhstan can be a driving force for a large part of recent aggregate fertility changes. During the Soviet period, there was officially sponsored inter-republic migration (Rakowska-Harmstone, 1977) and a big influx of Russians and other people of European origin within development programmes in industry and agriculture in Kazakhstan and the rest of Central Asia. The linguistic prevalence of the Russian language in all Soviet cities boosted Russian mobility and allowed Russians to perceive the entire Soviet Union as their motherland (Oka, 2007). Kazakhstan was a unique case among the Soviet republics because Russians and other people of European origin outnumbered the titular ethnicity for a long time. Figure 2 shows that it ethnically has been a predominantly Russian republic for a long time, culturally closer to Russia than to Central Asia.

After Kazakhstan gained independence, many Russians and other ethnic groups of European origin (German, Polish, Ukrainian, and others) emigrated from the country, while the new state at the same time initiated a programme of repatriation of ethnic Kazakhs from the countries they had migrated to during Soviet times, including China, Mongolia, Uzbekistan, Russia, Turkmenistan, Kyrgyzstan, Iran, Afghanistan, and Pakistan. According to recent official statistics, as of 2021, ethnic Kazakhs constituted 70.4% of the population in the country, while Russians remained the second largest ethnic group. (Kazakhstan ).

Relatedly, notable spatial variations and regional disparities can be observed within Kazakhstan. The Northern and Eastern regions, situated closer to Russia, exhibit a higher concentration of Russian and other Slavic populations, reflecting stronger cultural ties to Russia. On the other hand, the Southern and Western parts of Kazakhstan, which are in closer proximity to other Central Asian countries, show a stronger cultural connection to the Turkic and Muslim world.

Several studies (Aydıngün, 2007; Telebaev, 2003; Yerekesheva, 2020) also link the nation-building process after gaining independence to an increase in religiosity among ethnic Kazakhs who were searching for self-identification after the collapse of the Soviet Union. They point out that Kazakhs were using religion as a proxy for understanding how to be ‘a Kazakh’. The restoration of traditions, customs, and Muslim norms could thus affect family formation and fertility among ethnic Kazakhs.

## Hypotheses

In this study, recent parity-specific fertility developments in Kazakhstan are studied, including the assessment of possible differences in fertility trends by ethnicity and education. Based on the theoretical framework of fertility developments, the following hypotheses will be assessed:

1. If there is a reversal in the demographic fertility transition, there will be a gradual rise in fertility rates across all birth orders during the 2000s, with a particular emphasis on higher-order births.
2. If the decline in fertility in the 1990s was temporary and caused by the economic crisis, fertility would increase in the early periods of economic improvement but further stabilize at later stages.
3. If the Second Demographic Transition is developing in Kazakhstan, lower rates of first births and lower progressions to higher parities are expected for a) highly educated women due to their role as forerunners in other contexts, and b) ethnic Russian women due to potential similarities in fertility development with co-ethnics in other parts of the former Soviet Union.
4. In line with findings from other contexts, increasing rates of progression to parenthood among highly educated women are expected in periods of economic growth, while increasing rates of progression toward parenthood and subsequent births are expected among less educated women in periods of economic recession.
5. Decreasing rates of progression to third and fourth births are expected in general but especially among highly educated women, in line with the progression of the fertility transition.

## Data and Methods

### Data

Three rounds of the Kazakhstan Multiple Indicator Cluster Survey (MICS), collected in 2006, 2011, and 2015, have been used for analysis. The sample sizes were 14,719, 14,228, and 12,910 women for each round, respectively, with an average response rate of 98%. Only completed interviews, 41,243 (98.5% of the full sample), were used for the analysis.

MICS data does not contain complete fertility histories, and only the dates of the first and the last birth in a woman’s fertility history are included. However, the birth dates of all children under 18 who live in the household are known. The survey thus does not provide information on the exact birth dates of children who are not first or last, unless they live in the household. In addition, there is information about a mother’s identification number only for children under 18. For these reasons, the sample includes only the youngest cohorts to study higher-order births: women in the 2006 round were excluded if they gave birth to a first child before 1989, before 1994 for women surveyed in 2011, and before 1998 for women surveyed in 2015.

The sample is thus moderately selective based on the survival of children or cohabitation with the mother between the birth of the first and last child. Consequently, this implies that in cases where a woman's second child has passed away or left the household, and she has given birth three times, it becomes impossible to study her likelihood of a second birth due to the lack of information regarding the second child's date of birth. These exclusion criteria do not affect women who have not yet given birth to three or more children. Thus, the sample sizes for the analysis are as follows: first birth—41,242 women (no restrictions), second birth—16,920 women (or 93.6% of women from young cohorts that gave first birth not earlier than 17 years before an interview that allows us to analyse in-between births), third birth—10,274 (or 90.6% of women from young cohorts who have at least two children), and fourth birth—3821 (or 79% of women from young cohorts who have at least three children). The risk of first birth was analysed for 27,309 Kazakh women, 8953 Russian women, and 4980 women of other ethnicities. The risk of second birth was analysed for 11,121 Kazakh women, 3717 Russian women, and 2082 women of other ethnicities. The risk of third birth was analysed for 7462 Kazakh women, 1575 Russian women, and 1237 women of other ethnicities. The risk of fourth birth was analysed for 3197 Kazakh women, 223 Russian women, and 401 women of other ethnicities.

Moreover, for birth date information taken from the household roster (covering any births between the first and last child), the 2006 household dataset does not contain information on the month of birth other than the first and last births. Thus, the month of birth was randomly imputed for around 7% of cases for the study of second-birth risks and around 4% of cases for the study of third and fourth-birth risks.

To assess the reliability of the results based on MICS data, I compared the fertility estimates reported in the MICS final reports (2006, 2011, 2015) with estimates from the civil registration records. The TFR indicated in both the 2011 MICS report and the 2011 civil registration data is approximately 2.6. However, in the 2015 MICS report, the TFR was slightly higher at 3.0, compared to the TFR of 2.73 reported in the 2015 civil registration data. Previous studies in different contexts have discovered that the underestimation of single women in survey data leads to discrepancies between fertility estimates based on surveys and those based on civil registration records (Spoorenberg, 2014, 2017). I compared the number of never-married women in the 1999 and 2009 censuses (Statistics Agency, 2010) with the proportion in the pooled MICS data (2006, 2011, 2015). In the 1999 census, 29% of women aged 15–49 had never been married, whereas in the 2009 census, the percentage of never-married women in the same age group increased to 37%. The proportion of never-married women aged 15–49 in the pooled MICS data was 26%. Overall, a slight underrepresentation of single women aged 15–49 could be behind some discrepancies between survey-based fertility estimates and the estimated based on the civil registration data. Thus, the analysis may slightly overestimate the progressions to parenthood or subsequent births.

Similarly, I compared the ethnic composition collected in the MICS with the ethnic composition in population censuses 2009 and 2021 (Statistics Agency, 2010; Statistics Committee, 2022a, 2022b). Based on the 2009 population census in Kazakhstan, ethnic Kazakhs accounted for 63.1% of the population, while ethnic Russians comprised 23.7%. However, according to the 2021 population census, the proportion of ethnic Kazakhs increased to 70.4%, while the percentage of ethnic Russians decreased to 15.5%. Despite the pooled MICS data spanning 2006, 2011, and 2015, and the measurement of ethnicity based on the household head, the proportions it yields remain comparable: 66.3% of women are ethnic Kazakhs, and 21.7% are ethnic Russians. Overall, there seems to be no significant difference in the ethnic composition captured by the MICS data compared to the data collected during the two censuses.

Another possible limitation of using survey data to assess fertility estimates is the mass emigration of ethnic Russians from Kazakhstan after the collapse of the Soviet Union. The number of ethnic Russians decreased by 26.4% between the 1989 and 1999 censuses, by 15.3% between the 1999 and 2009 censuses, and by 21.4% between the 2009 and 2021 censuses (Statistics Committee, 2022a, 2022b). One potential bias in the MICS surveys, specifically regarding ethnic Russians, is that the cross-sectional sample of Kazakhstan may include individuals with different fertility behaviours compared to ethnic Russians who had previously resided in Kazakhstan but left the country before the survey data collection. It could be that childless ethnic Russians were more prone to emigrate from Kazakhstan than those with children, for example. In addition, the ethnic Russians who stayed in Kazakhstan were potentially more integrated into Kazakh culture. These possible sources of selection are a limitation, and the fertility estimates especially for ethnic Russians thus need to be interpreted with caution. In a study comparing fertility estimates based on Swedish register data, and a simulated scenario excluding emigrated individuals to mimic survey data, Andersson and Sobolev (2013) found that the exclusion of emigrated individuals had minimal impact on fertility measures. While the current study acknowledges the potential bias resulting from mass emigration of ethnic Russians from Kazakhstan, it does not address the extent to which fertility estimates would differ if Russians who had resided in Kazakhstan during specific periods but had moved before the survey were included.

#### Description of Variables

*Woman’s age* is the basic time factor to study the risk of first birth and it is a time-varying variable. The trajectory is followed from age 15 until the arrival of the first birth or the time of the interview, whichever comes first (the respondents consist of women aged 15–49, so there is no need to create an upper limit of 50).

*Duration since last birth* is the basic time factor to study the risk of second/third/fourth birth, and it is a time-varying variable. The trajectory is followed from the first/second/third birth until the arrival of the second/third/fourth birth or the time of the interview, whichever comes first.

*Age at last birth* is a time-constant variable to study second/third/fourth birth risks.

*Ethnicity* is one of the key predictors of the study, and it is a time-constant variable. The actual description of the variable in the raw dataset is the ethnic group of household head, and this is a limitation of the data. However, according to the 2009 census, out of all registered marriages, less than 4% of Kazakhs and 15% of Russians were involved in interethnic marriages (78% of Russians were not involved in interethnic marriages, while 7% were married to culturally close Ukrainians and Germans). The Assembly of People of Kazakhstan (a national body representing different ethnicities) reports that only 6% of marriages in 2017 were interethnic.

*Education* is measured using dynamic modelling of educational trajectories, following the advice of Hoem and Kreyenfeld (2006) to avoid anticipatory analyses of educational attainment and first-birth risks. Educational histories are reconstructed using variables that offer information about the highest educational level a respondent had achieved at the time of the interview. The reconstruction procedure assumes a trajectory of rigid educational progress with no breaks in studying, repeating school years, or postponing the entrance to a subsequent level. It traces respondents from the legal age of primary school entry (age 7) to their highest achieved educational level. The variable ‘the highest grade at that level’ helps to specify the exact number of years a respondent has spent at the highest attained educational level. A new academic year starts in September and ends the following May.

Primary school consists of 4 years of schooling and secondary school of 7 years (or 6 years, if the person started before 1987), but if a student chooses his/her subsequent level as a secondary vocational school instead of university, he/she only makes 5 years of secondary school; secondary vocational school consists of 3 additional years. Higher education can consist of 4–6 years (or more, if a doctoral degree is pursued). Before joining the Bologna educational system, students were supposed to study 5 years for a ‘specialist’ degree; nowadays it is 4 years for a bachelor’s degree and 2 additional years for a master’s degree. Information on the highest grade attained allows one to differentiate between the different levels. A time-varying binary variable was constructed to indicate periods in and out of education. The respondents are coded as being in education all the time before they attained the level reported in the interview. Thus, the variable ‘education’ is time-varying and consists of 5 levels: in education, none/primary/not completed secondary, completed secondary, secondary specialized, and higher. This time-varying variable is used in the first-birth models.

For the analyses of the progressions to second, third, and fourth parities, the educational attainment at the time of the interview is used, based on the assumption that women rarely continue their education after entering parenthood.

*Calendar year* is a time-variant covariate and the main variable of interest in this study. When using a period approach, changes in behaviour are observed for synthetic cohorts over time, ‘which is an imaginary group of people who experience, hypothetically, the demographic conditions of that period throughout life’ (Willmoth, 2005, p. 234). Women contribute to the period estimates as they pass through different years at each given parity. The following calendar year groups are used in the study of first-birth risks: 1971–1980, 1981–1990, 1991–1995, 1996–2000, 2001–2005, 2006–2010, and 2011–2015. The first two periods cover the Soviet era and are split into two periods to see the dynamics in reproductive behaviour over this relatively long period, including the 1980s with its pro-natalist policies; the next two periods cover the time of economic crisis, and the last three periods cover the time of economic recovery. The periods for the study of second/third/fourth-birth risks are slightly different because of the peculiarities of the dataset and the possibility of studying only the more recent cohorts for higher-order births. In this case, calendar years are aggregated into 1989–1994, 1995–2000, 2001–2005, 2006–2010, and 2011–2015. The last years of the Soviet period had to be combined with the first years of the economic crisis.

Summary statistics of exposures and occurrences by every variable and each parity are presented in Tables 5, 6, 8, and 10 in Appendix.

### Methods

To analyse first-, second-, third-, and fourth-birth rates, I apply event history analysis, which is useful when analysing time-dependent processes and allows the characteristics of the respondent to change over time. I present findings as parity-specific relative risks of giving birth during 1971–2015, adjusted for the age of a woman or duration since the last birth, ethnicity, and education. Concerning second, third, and fourth births, mothers are excluded from a given parity sample if they had multiple births the first, second, or third time, respectively.

## Results

### First-Birth Risks

Table 1 shows that the first birth risks are the highest at the relatively young age of 21–26 and then gradually decrease with age. Despite the expectation of delayed parenthood among population subgroups that may be in a more advanced stage of the second demographic transition, it is observed that ethnic Russian women tend to become mothers at an earlier age compared to ethnic Kazakh women. Education shows a negative gradient with the timing of first births; the higher the educational level achieved, the lower the risks of first birth. As regards the calendar period, relative risks of first birth increased in 1981–1990, in line with the pronatalist policies of the late Soviet period. Additionally, during the initial years of independence, there was an upward trend observed, despite the presence of an ongoing economic crisis. During the later stages of the economic crisis, a decline in relative first-birth risks can be observed, but the risks were still higher than in the earliest period. The decline continued in the first period of economic growth (2001–2005), but in the latest periods of economic recovery, the risks of first birth gradually increased again.

**Table 1:** Relative risks of first birth for women in Kazakhstan by age, ethnicity, education and calendar period, 1971–2015

	Relative risk	S. E	*P* > *z*
*Age*			
15–17	0.08	0.00	0.000
18–20	0.57	0.01	0.000
21–23	1.00		
24–26	0.91	0.02	0.000
27–29	0.70	0.02	0.000
30–32	0.48	0.02	0.000
33–35	0.39	0.02	0.000
36–38	0.20	0.02	0.000
39–41	0.14	0.02	0.000
42+	0.04	0.01	0.000
*Ethnicity*			
Kazakh	1.00		
Russian	1.19	0.02	0.000
Other	1.14	0.02	0.000
*Education*			
In education	0.37	0.01	0.000
None/primary/not completed secondary	0.91	0.05	0.097
Secondary	1.00		
Secondary vocational	0.84	0.01	0.000
Higher	0.72	0.01	0.000
*Calendar period*			
1971–1980	1.00		
1981–1990	1.28	0.04	0.000
1991–1995	1.48	0.05	0.000
1996–2000	1.14	0.04	0.000
2001–2005	1.08	0.04	0.026
2006–2010	1.19	0.04	0.000
2011–2015	1.35	0.06	0.000
_cons	0.02	0.00	0.000
# of subjects	41,179		
# of occurrences	28,338		
Time at risk	3,873,261		
Log likelihood	− 30313.076		
Prob > chi2	0.0000		

Table 2 shows the first-birth estimates, using the Kaplan–Meier function at specific ages, calculated as synthetic cohorts for the calendar periods by ethnicity, which can be interpreted as the share of women at a given age that would become a mother given the transition rates in that period. It can be observed that the difference between the two ethnicities in first-birth estimates by age 25 was fairly small in the post-Soviet era, but larger in the period characterized by the Soviet pronatalist policies. Almost all women enter parenthood by age 35 (over 90%) and there is no trend over time to indicate increased childlessness, nor increasing differences between the two ethnicities.

**Table 2:** First-birth estimates for Kazakh and Russian women in Kazakhstan across six time periods, Kaplan–Meier function, by age 25 and 35

	Kazakh		Russian		All ethnicities	
	Age 25	Age 35	Age 25	Age 35	Age 25	Age 35
1981–1990	0.67	–	0.81	–	0.71	–
1991–1995	0.73	0.94	0.76	0.92	0.75	0.93
1996–2000	0.62	0.88	0.66	0.88	0.64	0.88
2001–2005	0.59	0.87	0.61	0.87	0.60	0.87
2006–2010	0.62	0.89	0.60	0.88	0.62	0.89
2011–2015	0.65	0.91	0.62	0.92	0.66	0.92

KM function values at specific ages. The period 1971–1980 is not included because there are no women over the age of 20 contributing to this synthetic cohort. Age 35 is not included for 1981–1990 because there were no women over the age of 30 contributing to this synthetic cohort.

**Fig. 3:**
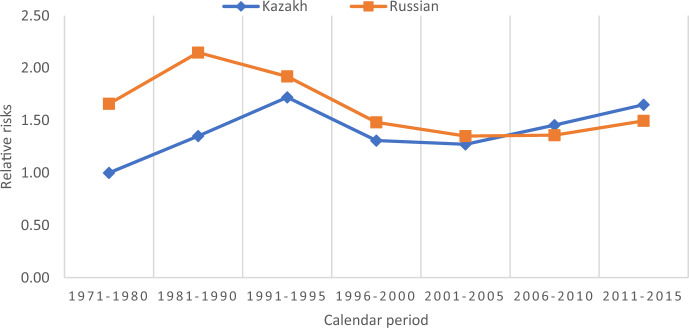
Relative risks of first birth, interaction between ethnicity and calendar period (reference Kazakh 1971–1980), controlling for all other factors. *Note* The interaction is significant according to the likelihood-ratio test. LR chi2(12) = 199.37. Prob > chi2 = 0.000. Interaction also improves the model fit according to the AIC/BIC criteria

Figure 3 shows the interaction between ethnicity and calendar period in first-birth risks. Both Kazakhs and Russians experience an increase in relative risks in the 1981–1990 period, while during the first years of independence, there was a decrease in relative risks among Russians but an increase among Kazakhs. In the later period of the 1990s and the early 2000s, the relative risks of first birth decreased for Kazakhs and Russians alike. It was followed by an increase for both ethnic groups in the latter two periods, which was somewhat more pronounced among Kazakhs whose relative risks of first birth became even higher than among Russians. Taken together, a convergence of first-birth risks of the two ethnicities over time is observed.

**Fig. 4:**
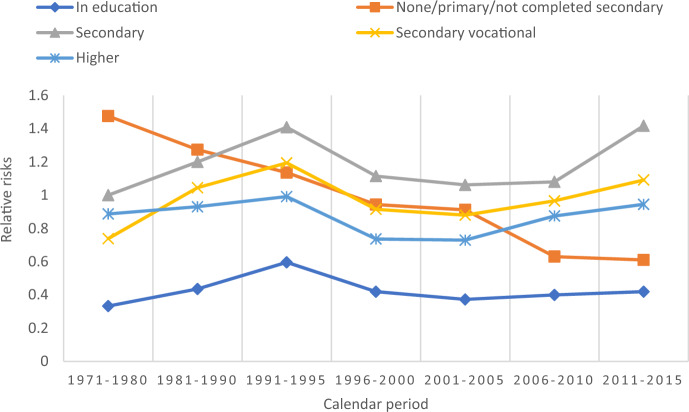
Relative risks of first birth, interaction between education and calendar period (reference completed secondary 1971–1980), controlling for all other factors. *Note* The interaction is significant according to the likelihood-ratio test. LR chi2(24) = 57.18. Prob > chi2 = 0.000. Interaction also improves the model fit according to AIC criterion

Figure 4 shows the interaction between education and calendar period. Apart from the none/primary/non-completed secondary category that gradually decreased its first-birth risks across periods, quite a uniform trend across the other four categories is observed. So, the relative risks of first birth first increased in the 1981–1990 and 1991–1995 periods among the other four educational categories and then uniformly decreased in the 1996–2000 and 2001–2005 periods. This is followed by a subsequent increase in the 2006–2010 and 2011–2015 periods among the same four educational categories.

Additionally, a three-way interaction between ethnicity, education, and calendar period (see Appendix, Fig. 6) did not show any contrasting pattern of relative risks of first birth across the periods between the two ethnicities depending on educational level.

### Second-Birth Risks

The relative risks of second birth for most of the control variables demonstrate expected relationships (Table 3): second-birth rates were lower when the age at the first birth was higher; the higher the educational level achieved, the lower the second-birth rates. The transition rate increased within the first three years after the first child was born and decreased after this point. The second-birth risks are around two and a half times higher for Kazakh women than for Russian women. The calendar period shows that second parity transitions were stable during the 1990s, while there was a gradual increase in the relative risks of second birth during the post-Socialist periods of economic recovery.

**Table 3:** Relative risks of second/third/fourth birth for women in Kazakhstan, by duration since previous birth, age at previous birth, ethnicity, education, and calendar period

	Second-birth risks			Third-birth risks			Fourth-birth risks		
	Relative risk	S.E	*P* > *z*	Relative risk	S.E	*P* > *z*	Relative risk	S.E	*P* > *z*
*Duration since the last birth*									
0–1 years since first child born	0.51	0.01	0.000	0.44	0.02	0.000	0.53	0.04	0.000
2–3 years since first child born	1.00			1.00			1.00		
4–5 years since first child born	0.76	0.02	0.000	1.03	0.05	0.564	0.79	0.07	0.005
6–7 years since first child born	0.63	0.02	0.000	0.93	0.05	0.186	0.70	0.08	0.002
8–9 years since first child born	0.48	0.02	0.000	0.67	0.05	0.000	0.63	0.11	0.007
10 and more years since first child born	0.26	0.02	0.000	0.49	0.04	0.000	0.40	0.13	0.007
*Age at last birth*									
19 or less	0.96	0.03	0.113	0.76	0.11	0.056	1.54	0.90	0.461
20–24	1.00			1.00			1.00		
25–29	0.82	0.02	0.000	0.85	0.03	0.000	0.84	0.07	0.045
30–34	0.54	0.03	0.000	0.57	0.03	0.000	0.59	0.06	0.000
35 +	0.22	0.03	0.000	0.28	0.04	0.000	0.31	0.05	0.000
*Ethnicity*									
Kazakh	1.00			1.00			1.00		
Russian	0.38	0.01	0.000	0.24	0.02	0.000	0.39	0.07	0.000
Other	0.70	0.02	0.000	0.61	0.03	0.000	0.74	0.07	0.003
*Education*									
None/primary/some secondary	0.95	0.05	0.275	1.05	0.08	0.507	0.90	0.13	0.463
Secondary	1.00			1.00			1.00		
Secondary vocational	0.76	0.02	0.000	0.71	0.03	0.000	0.87	0.06	0.042
Higher	0.69	0.02	0.000	0.66	0.03	0.000	0.74	0.06	0.000
*Calendar period*									
1989–1994	1.00			1.00					
1995–2000 (1992–2000 for 4th birth risks)	0.96	0.04	0.372	0.92	0.12	0.535	1.00		
2001–2005	1.09	0.05	0.048	0.91	0.12	0.474	1.05	0.11	0.66
2006–2010	1.39	0.06	0.000	1.49	0.19	0.002	1.28	0.13	0.013
2011–2015	1.64	0.08	0.000	1.42	0.19	0.007	1.21	0.13	0.075
_cons	0.03	0.00	0.000	0.01	0.00	0.000	0.02	0.00	0.000
# of subjects	16,890			10,230			3637		
# of occurrences	10,219			3747			1207		
Time at risk	928,047			588,916			155,386		
Log likelihood	− 18054.52			− 7946.4824			− 2735.932		
Prob > chi2	0.0000			0.0000			0.0000		

Table 4 displays the transitions to a second birth among women, calculated for synthetic cohort calendar periods by ethnicity, using the Kaplan–Meier function. This information can be interpreted as the proportion of women at a specific duration since their first birth who had a second child, based on the transition rates observed during that particular time period. The second-birth estimates at 4 years since the first birth were uniformly decreasing in 1995–2000, but at different levels for the two ethnicities. The estimates for ethnic Kazakhs were almost two and half times higher by 2001, and the difference increased further in the later periods to become up to three times higher than the estimates for ethnic Russians. At 6 years since the first birth, the estimates were stable for ethnic Russians until 2006. After 2006 there was a slight increase in second-birth estimates among Russian mothers. The respective estimates for Kazakh women show that after some decrease in the late 1990s and early 2000s, there was a gradual increase in the subsequent periods. Almost all Kazakh one-child mothers in the most recent period had a second child by 10 years after the first birth (86%), and there is a clear trend over time to indicate an increased progression toward second births among ethnic Kazakhs. A trend of increased progression to second births is also observed among ethnic Russians, and 61% of Russian one-child mothers then had a second child within 10 years of the first birth.

**Table 4:** Transition to second/third/fourth birth of women in Kazakhstan, by ethnicity across four time periods, Kaplan–Meier estimates

	Kazakh			Russian			All ethnicities		
	4 years	6 years	10 years	4 years	6 years	10 years	4 years	6 years	10 years
*Transition to a second birth*									
1989–1994	0.58	0.74		0.27	0.34		0.51	0.64	
1995–2000	0.51	0.65	0.77	0.21	0.32	0.46	0.56	0.64	0.68
2001–2005	0.53	0.67	0.8	0.19	0.32	0.5	0.44	0.58	0.73
2006–2010	0.61	0.75	0.85	0.23	0.38	0.58	0.51	0.65	0.78
2011–2015	0.69	0.79	0.86	0.24	0.4	0.61	0.58	0.69	0.81
*Transition to a third birth*									
1989–1994	0.31			0.07			0.25		
1995–2000	0.28	0.4	0.54	0.1	0.14	0.17	0.25	0.36	0.47
2001–2005	0.25	0.38	0.56	0.09	0.13	0.2	0.22	0.34	0.51
2006–2010	0.35	0.55	0.72	0.1	0.14	0.29	0.3	0.47	0.64
2011–2015	0.33	0.51	0.67	0.05	0.14	0.25	0.27	0.43	0.58
*Transition to a fourth birth*									
1992–2000	0.3	0.43		0.26			0.29	0.41	
2001–2005	0.34	0.45	0.6	0.29	0.37		0.32	0.43	0.57
2006–2010	0.4	0.51	0.7	0.19	0.3	0.3	0.39	0.5	0.68
2011–2015	0.33	0.49	0.66	0.07	0.1	0.2	0.3	0.47	0.63

KM function values at specific duration since previous birth

Additionally, an interaction between ethnicity and calendar period did not improve the model fit (see Appendix, Fig. 8). These results show that even though the two main ethnicities in Kazakhstan have very different initial levels of progression to a second birth, ups and downs in the second-birth rates across these periods were shared in parallel by both groups.

The interaction between education and calendar period (see Appendix, Fig. 9) also did not improve the model fit according to BIC criteria, thus also not revealing any specific responses by educational level. Additionally, a three-way interaction between ethnicity, education, and calendar period (see Appendix, Fig. 10) did not show any contrasting pattern of relative risks of second birth across the periods between the two ethnicities depending on educational level.

### Third-Birth Risks

Table 3 (column 3) shows that Kazakh two-child mothers are four times more prone to give birth to a third child than Russian women. The relative risks for most of the control variables demonstrate expected relationships: third-birth rates are lower when the age at second birth is higher; the higher the educational level achieved, the lower the third-birth rates. The transition rate was the highest within 2–7 years after the second child was born, and decreased after this point. The relative risks were higher in 2006–2010 and 2011–2015 than in the previous years.

Table 4 shows the transitions to third births among women, using the Kaplan–Meier function, at selected durations since second birth, calculated for synthetic cohort calendar periods by ethnicity. The third-birth estimates at 4 years since second birth were decreasing among ethnic Kazakhs in the late 1990s and early 2000s, with increases and stabilization in later periods. The corresponding estimates for Russians were stable at far lower levels until 2011 when they decreased even further. By 6 years after second birth, only 14% of two-child Russian mothers had a third child, and this pattern was stable over time. In contrast, more than half of Kazakh two-child mothers had a third child within 6 years of their second birth, and an increase can be observed in this level in the most recent periods. Around two-thirds of Kazakh two-child mothers had a third child by 10 years after their second birth and a gradual increase is observed in this level over time. The corresponding number for Russian two-child mothers is just 25%. The third-birth rates increased also for this group, albeit at a much lower level.

The interaction between ethnicity and calendar period (see Appendix, Fig. 12) did not improve the model fit. Similar non-significant results were found for the model with an interaction between education and calendar period (see Appendix, Fig. 13). Additionally, a three-way interaction between ethnicity, education, and calendar period (see Appendix, Fig. 14) did not show any significant contrasting pattern in the relative risks of third birth.

### Fourth-Birth Risks

Table 3 (column 4) shows that Kazakh three-child mothers are 2.5 times more prone to give a birth to a fourth child than Russian women. The relative risks for most of the control variables demonstrate expected relationships: fourth-birth rates were lower when the age at the third birth was higher; a negative gradient is also found for the duration since third birth and educational level.

Table 4 shows the transitions to fourth births among women, using the Kaplan–Meier function, at selected durations since third birth, calculated for the calendar periods by ethnicity. The fourth-birth estimates at 4 years since the third birth were stable over time among ethnic Kazakhs except for some increases in 2006–2010. A decreasing trend over time can be seen for ethnic Russians at the same duration since third birth. A similar gradual decrease is observed for Russians at 6 and 10 years since third birth for the periods where data are available. Thus, only 20% of Russian mothers of three children gave birth to a fourth child within 10 years of the third birth. For ethnic Kazakhs, increased transition rates are observed since 2006 at the more extended durations since third births. Two-thirds of Kazakh mothers of three children had given birth to a fourth child by 10 years after their third birth.

**Fig. 5:**
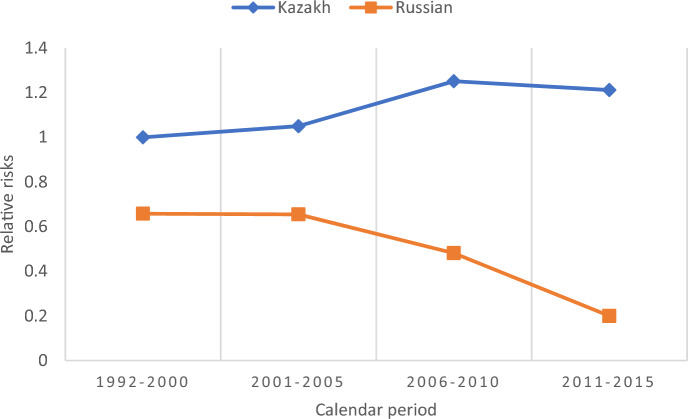
Relative risks of fourth birth, interaction between ethnicity and calendar period (reference Kazakh 1992–2000), controlling for all other factors. *Note* The interaction is significant according to the likelihood-ratio test. LR chi2 (6) = 13.95. Prob > chi2 = 0.0302. The interaction also improves the model fit according to the AIC criterion

**Fig. 6:**
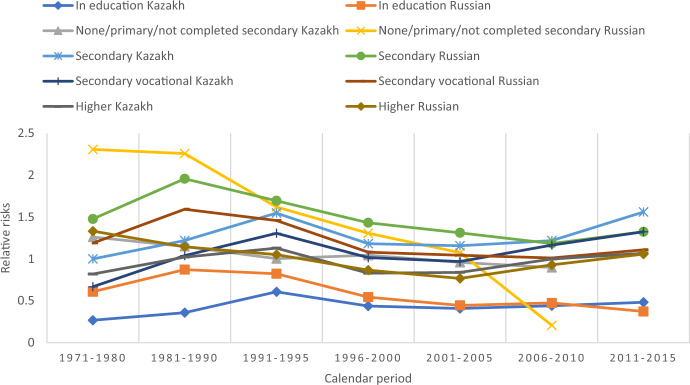
Relative risks of first birth, three-way interaction between ethnicity, education and calendar period (reference Kazakh completed secondary 1971–1980), controlling for all other factors. *Note* The interaction does not improve the model fit according to the BIC criterion

**Fig. 8:**
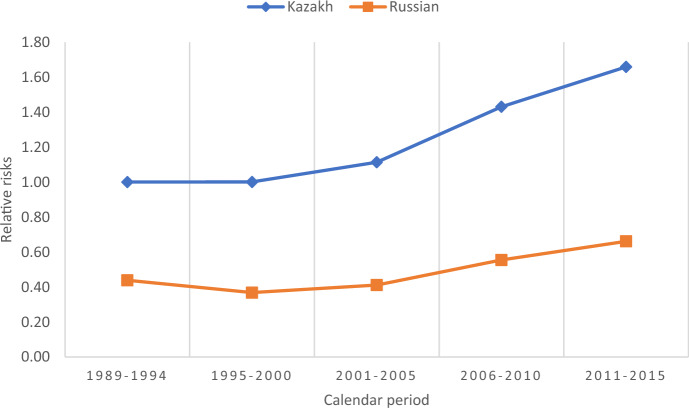
Relative risks of second birth, interaction between ethnicity and calendar period (reference Kazakh 1989–1994), controlling for all other factors. *Note* The interaction does not improve the model fit according to the AIC/BIC criteria

**Fig. 9:**
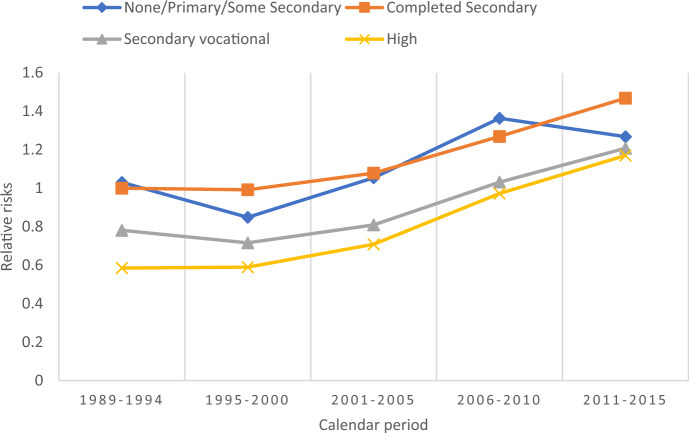
Relative risks of second birth, interaction between education and calendar period (reference completed secondary 1989–1994), controlling for all other factors. *Note* The interaction does not improve the model fit according to the BIC criterion

**Fig. 10:**
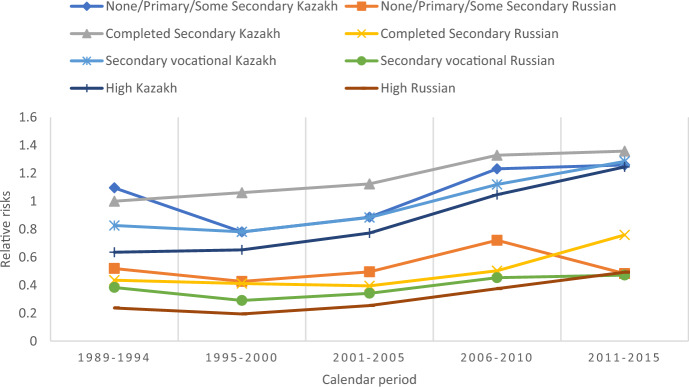
Relative risks of second birth, three-way interaction between ethnicity, education and calendar period (reference Kazakh completed secondary 1989–1994), controlling for all other factors. *Note* The interaction does not improve the model fit according to the AIC/BIC criteria

**Fig. 12:**
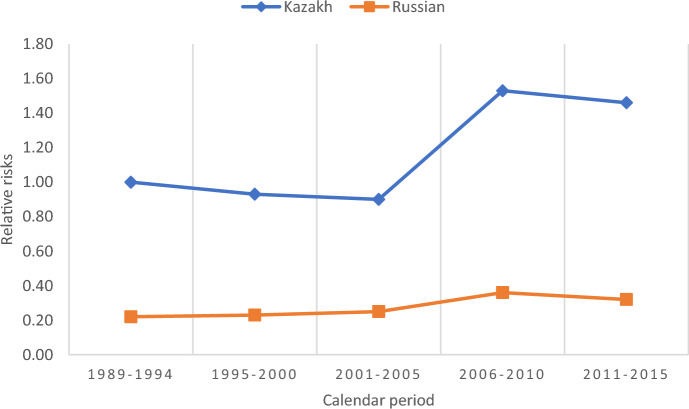
Relative risks of third birth, interaction between ethnicity and calendar period (reference Kazakh 1989–1994), controlling for all other factors. *Note* The interaction does not improve the model fit according to the AIC/BIC criteria

**Fig. 13:**
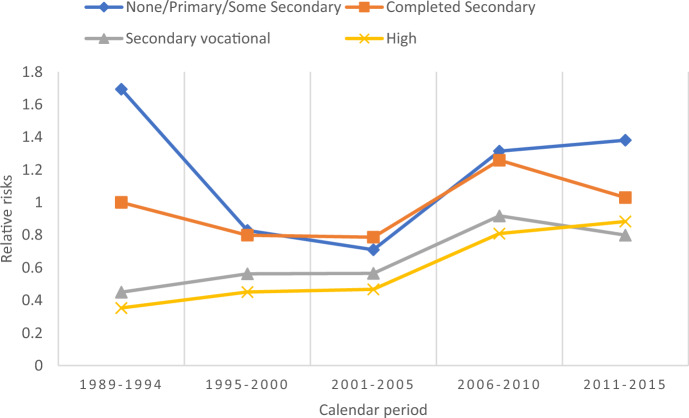
Relative risks of third birth, interaction between education and calendar period (reference completed secondary 1989–1994), controlling for all other factors. *Note* The interaction does not improve the model fit according to the AIC/BIC criteria

**Fig. 14:**
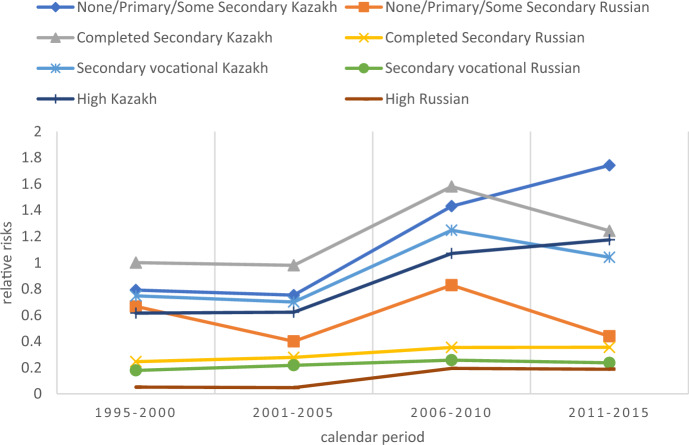
Relative risks of third birth, three-way interaction between ethnicity, education and calendar period (reference Kazakh completed secondary 1989–1994), controlling for all other factors. *Note* The interaction does not improve the model fit according to the AIC/BIC criteria

Figure 5 shows the relative risks of fourth birth in the model with an interaction between ethnicity and calendar period. A stability of relative risks for both Kazakhs and Russians is observed, although at different levels, until 2001–2005, followed by an increase in the calendar period 2006–2010 among the Kazakhs. Among Russians, the earlier stability was followed by a gradual decrease in four-birth rates in 2006–2015.

**Fig. 15:**
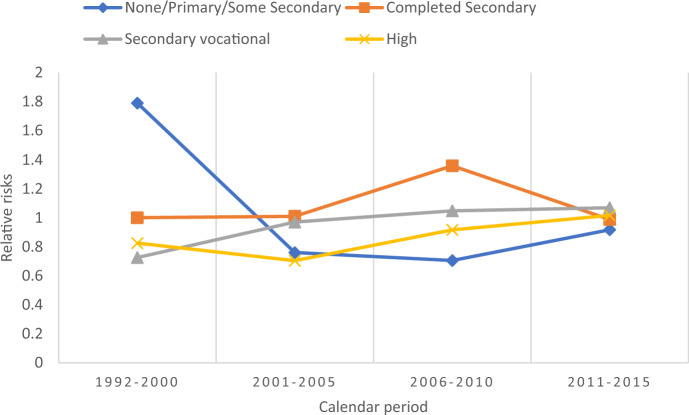
Relative risks of fourth birth, interaction between education and calendar period (reference completed secondary 1992–2000), controlling for all other factors. *Note* The interaction does not improve the model fit according to the AIC/BIC criteria

**Fig. 16:**
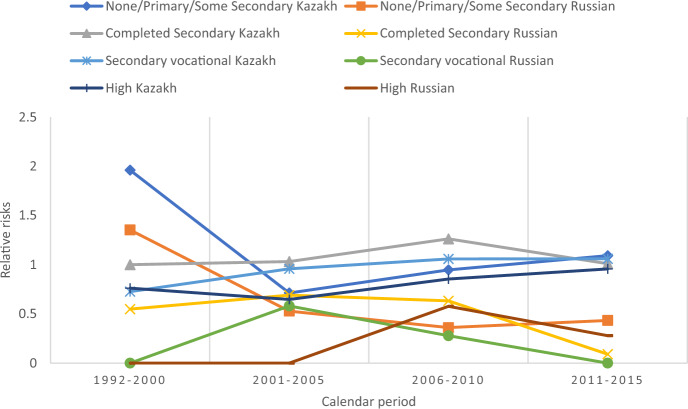
Relative risks of fourth birth, three-way interaction between ethnicity, education and calendar period (reference Kazakh completed secondary 1992–2000), controlling for all other factors. *Note* The interaction does not improve the model fit according to the AIC/BIC criteria

The interaction between education and calendar period (see Appendix, Fig. 15) did not improve the model fit. Additionally, a three-way interaction between ethnicity, education, and calendar period (see Appendix, Fig. 16) did not show any further significant patterns of fourth-birth risks across all three categories.

## Discussion and Conclusions

This study aimed to investigate whether the previously observed fertility increases in Kazakhstan during the early 2000s were a temporary phenomenon or indicative of a sustained period of high fertility levels. Additionally, the study assessed whether the pattern of fertility recuperation was universal or specific to particular subgroups of the population, taking into consideration any variations in behaviour and outcomes based on ethnicity and education.

In line with hypothesis 1, that the fertility part of Kazakhstan’s demographic transition may have gone into reverse, a gradual increase in fertility rates was observed at all birth orders after the turn of the millennium. This is especially striking for higher-order births, for which fertility developments can most convincingly be linked to changes in the progress of the demographic transition. Hypothesis 2, which posited stabilization after an initial increase during early periods of economic improvement, is not supported. This is evident as a gradual and continuous increase is observed in later periods, even in the absence of additional economic improvements. It is worth noting that while there was economic growth in terms of GDP, this might not necessarily correspond to improvements in individual real income. Furthermore, there is no apparent overall decline in the rate of first births during the study period, which could have indicated the influence of fertility postponement and been considered as evidence of progress towards a second demographic transition. There is some indication of fertility postponement among ethnic Russian women in the early twenty-first century. However, this trend later stabilized, and a continuous postponement is not observed for this group. Specifically, there is no indication of a continuous trend towards postponing motherhood among the majority group of ethnic Kazakh women.

A pattern of very low progression rates to second and third births among ethnic Russian women could be seen as some evidence of progress toward the second demographic transition. However, these progression rates did not show any evidence of decline during the period under analysis. Thus, hypothesis 3b is not supported. The estimates for ethnic Kazakhs indicate increasing rates for all higher-order births, which is a development contrary to what would typically be expected from the classical demographic transition. Nevertheless, the trends by ethnicity are quite similar even though Kazakh and Russian women have entirely different levels of parity progressions. This suggests that contextual factors are at play in shaping fertility change.

Business cycles and the provision of social policies may impact decisions regarding childbearing, and it would be reasonable to expect that women with varying levels of educational attainment would respond differently to changes in these factors. However, contrary to hypotheses 3a, hypothesis 4, and hypothesis 5, the results did not reveal any educational trend differentials in parity-specific fertility across the periods of the study. Although the risks were at different levels for educational groups, this homogeneity could indicate that childbearing still is an integral part of society in Kazakhstan, and even highly educated women are still eager to progress to higher-order births. It could also be seen as support for the notion of a broad-based re-traditionalization of the society.

This study extends the literature on different paths of demographic change and the possibility of reversals of fertility transitions to the context of post-Soviet Central Asia. As Burger and DeLong (2016) argue, trend reversals could be driven by changes in cultural norms that are not always necessarily moving towards more modernist values as in more developed countries. Thus, the Kazakh case may be closer to the experience of, for example, Iran after the Islamic Revolution, when the demographic transition stalled and even temporarily went into reverse (Aghajanian, 1991). It may also resemble patterns in other oil-rich countries in the Middle East where fertility patterns do not necessarily match economic development (Hakimian, 2006; Omran & Roudi, 1993).

The case study of Kazakhstan, as a predominantly Muslim country, may also contribute to the literature on Muslim countries as a case where public gender equality is high in relation to women’s education as well as labour force participation. Thus, the so-called MENA paradox of a mismatch between high educational attainment and low labour force participation (Buyukkececi & Engelhardt, 2021) as a possible explanation for high fertility does not apply to Kazakhstan. Instead, the paradox deepens, as women in Kazakhstan are both highly educated and participating in the labour market to a high extent, yet maintain relatively high fertility.

The limitations of this study are driven by the characteristics of the available data. There is a slight selectivity in the sample of survey respondents with respect to ever being married, as well as selectivity based on the survival of children and co-residence with children in-between the first and last birth, which limits the sample to more recent birth cohorts. Panel data and more accurate measures of ethnicity, religion, and time-varying data on rural/urban residence would be beneficial for future research.

Future research may explore the role of religion in keeping fertility at high levels. Patterns of parity-specific contraceptive use could also be studied to assess the change in contraceptive use and how this may have contributed to the trend reversal that is observed in fertility. If better time-varying data become available in the future, it will be worth exploring the effects of income changes as well as family policy designs on parity progressions to assess the role of widening wealth inequalities and dependence on social support in fertility change.

In sum, the study provides new evidence of a sustained fertility increase in Kazakhstan during the 2000s that is associated with a genuine increase in birth rates across all birth orders, ethnicities, and educational groups. Kazakhstan appears to be an example of a reversed fertility transition with increasing progressions to higher-order births and little fertility postponement. The study also suggests that contextual factors that affect all sectors of Kazakhstani society are at play in determining childbearing behaviour. The parity-specific trends are remarkably similar for ethnic and educational groups that otherwise have had different levels and patterns of childbearing.

## Figures and Tables

**Fig. 7:**
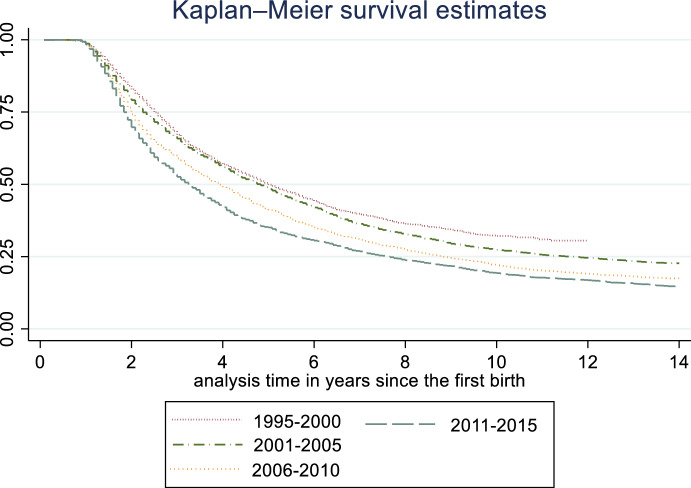
Second birth estimates for synthetic cohorts of one-child mothers in Kazakhstan, Kaplan–Meier estimates

**Fig. 11:**
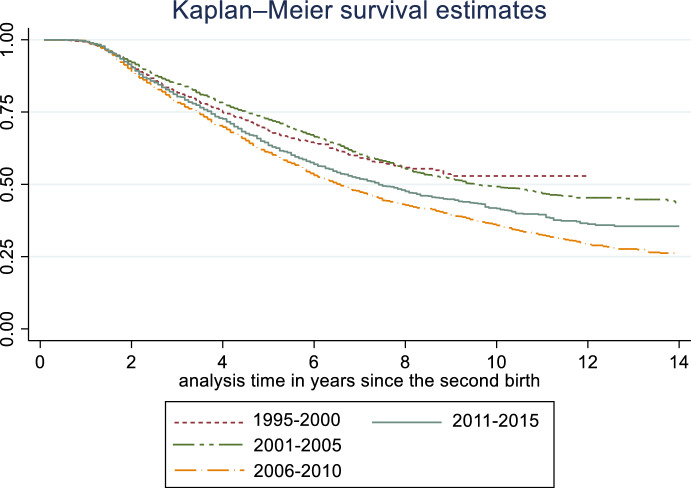
Third birth estimates for synthetic cohorts of two-child mothers in Kazakhstan, Kaplan–Meier estimates

## Data Availability

Data was obtained from the UNICEF Multiple Indicator Cluster Survey’s website: https://mics.unicef.org/surveys.

## Appendix

See Figs. 6, 7, 8, 9, 10, 11, 12, 13, 14, 15, 16 and Tables 5, 6, 7, 8, 9, 10, 11.

**Table 5:** Exposure distributions for all variables to the study of first birth risks

	Person-month	Occurrences	Rate	[95% Conf interval]	
*Age*					
15–17	1,405,088	1212	0.001	0.001	0.001
18–20	1,120,335	9084	0.008	0.008	0.008
21–23	641,573	10,046	0.016	0.015	0.016
24–26	310,331	4799	0.015	0.015	0.016
27–29	159,782	1896	0.012	0.011	0.012
30–32	90,899	735	0.008	0.008	0.009
33–35	57,412	372	0.006	0.006	0.007
36–38	37,469	122	0.003	0.003	0.004
39–41	25,078	56	0.002	0.002	0.003
42 +	25,294	16	0.001	0.000	0.001
*Ethnicity*					
Kazakh	2,619,154	18,347	0.007	0.007	0.007
Russian	815,451	6449	0.008	0.008	0.008
Other	438,656	3542	0.008	0.008	0.008
*Education*					
In education	1,548,966	3386	0.002	0.002	0.002
None/primary/not completed secondary	42,164	335	0.008	0.007	0.009
Secondary	1,045,468	10,015	0.010	0.009	0.010
Secondary vocational	750,384	9074	0.012	0.012	0.012
Higher	486,279	5528	0.011	0.011	0.012
*Calendar period*					
1971–1980	271,974	980	0.004	0.003	0.004
1981–1990	939,899	7128	0.008	0.007	0.008
1991–1995	569,771	5396	0.009	0.009	0.010
1996–2000	630,392	4512	0.007	0.007	0.007
2001–2005	747,454	4864	0.007	0.006	0.007
2006–2010	508,843	3662	0.007	0.007	0.007
2011–2015	204,928	1796	0.009	0.008	0.009
Total	3,873,261	28,338	0.007	0.007	0.007

**Table 6:** Exposure distributions for all variables for the study of second birth risks

	Person-month	Occurrences	Rate	[95% Conf interval]	
*Duration since the first birth*					
0–1 years since first child born	362,437	3310	0.009	0.009	0.009
2–3 years since first child born	214,754	3620	0.017	0.016	0.017
4–5 years since first child born	132,039	1634	0.012	0.012	0.013
6–7 years since first child born	85,428	870	0.010	0.010	0.011
8–9 years since first child born	57,230	444	0.008	0.007	0.009
10 and more years since first child born	76,159	341	0.004	0.004	0.005
*Age at first birth*					
19 or less	139,631	1554	0.011	0.011	0.012
20–24	486,365	5957	0.012	0.012	0.013
25–29	212,110	2171	0.010	0.010	0.011
30–34	70,809	474	0.007	0.006	0.007
35 +	19,132	63	0.003	0.003	0.004
*Ethnicity*					
Kazakh	535,694	7428	0.014	0.014	0.014
Russian	276,535	1564	0.006	0.005	0.006
Other	115,818	1227	0.011	0.010	0.011
*Education*					
None/primary/not completed secondary	36,717	431	0.012	0.011	0.013
Secondary	258,253	3485	0.013	0.013	0.014
Secondary vocational	302,573	3026	0.010	0.010	0.010
Higher	330,504	3277	0.010	0.010	0.010
*Calendar period*					
1989–1994	68,056	700	0.010	0.010	0.011
1995–2000	211,548	2140	0.010	0.010	0.011
2001–2005	296,922	3054	0.010	0.010	0.011
2006–2010	233,380	2781	0.012	0.011	0.012
2011–2015	118,141	1544	0.013	0.012	0.014
Total	928,047	10,219	0.011	0.011	0.011

**Table 7:** Second birth estimates for Kazakh and Russian women in Kazakhstan across five time periods, Kaplan–Meier estimates with confidence intervals

	Kazakh				Russian			
	Failure function	Std. error	[95%	conf.int.]	Failure function	Std. error	[95% Conf interval]	
*1989–1994*								
4 years since first birth	0.58	0.02	0.55	0.62	0.27	0.03	0.22	0.34
6 years since first birth	0.74	0.03	0.68	0.79	0.34	0.04	0.27	0.43
*1995–2000*								
4 years since first birth	0.51	0.01	0.49	0.53	0.21	0.01	0.18	0.24
6 years since first birth	0.65	0.01	0.63	0.67	0.32	0.02	0.28	0.35
10 years since first birth	0.77	0.01	0.74	0.79	0.46	0.03	0.41	0.51
*2001–2005*								
4 years since first birth	0.53	0.01	0.51	0.55	0.19	0.01	0.17	0.21
6 years since first birth	0.67	0.01	0.65	0.69	0.32	0.02	0.29	0.35
10 years since first birth	0.80	0.01	0.79	0.82	0.50	0.02	0.46	0.53
*2006–2010*								
4 years since first birth	0.61	0.01	0.59	0.63	0.23	0.02	0.20	0.26
6 years since first birth	0.75	0.01	0.73	0.76	0.38	0.02	0.35	0.41
10 years since first birth	0.85	0.01	0.83	0.86	0.58	0.02	0.54	0.62
*2011–2015*								
4 years since first birth	0.69	0.01	0.66	0.71	0.24	0.02	0.20	0.29
6 years since first birth	0.79	0.01	0.76	0.81	0.40	0.03	0.35	0.45
10 years since first birth	0.86	0.01	0.84	0.88	0.61	0.03	0.56	0.66

There are no women with spacing over 6 years contributing to the 1989–1994 synthetic cohort

**Table 8:** Exposure distributions for all variables for the study of third birth risks

	Person-month	Occurrences	Rate	[95% Conf interval]	
*Duration since the second birth*					
0–1 years since second child born	219,735	845	0.004	0.004	0.004
2–3 years since second child born	148,608	1272	0.009	0.008	0.009
4–5 years since second child born	95,518	829	0.009	0.008	0.009
6–7 years since second child born	59,089	462	0.008	0.007	0.009
8–9 years since second child born	35,608	204	0.006	0.005	0.007
10 and more years since second child born	30,358	135	0.004	0.004	0.005
*Age at second birth*					
19 or less	10,343	52	0.005	0.004	0.007
20–24	211,701	1627	0.008	0.007	0.008
25–29	245,780	1591	0.006	0.006	0.007
30–34	96,666	420	0.004	0.004	0.005
35 +	24,426	57	0.002	0.002	0.003
*Ethnicity*					
Kazakh	413,559	3152	0.008	0.007	0.008
Russian	102,267	209	0.002	0.002	0.002
Other	73,090	386	0.005	0.005	0.006
*Education*					
None/primary/not completed secondary	24,195	177	0.007	0.006	0.008
Secondary	198,229	1551	0.008	0.007	0.008
Secondary vocational	184,013	998	0.005	0.005	0.006
Higher	182,479	1021	0.006	0.005	0.006
*Calendar period*					
1989–1994	13,993	65	0.005	0.004	0.006
1995–2000	103,554	583	0.006	0.005	0.006
2001–2005	189,146	996	0.005	0.005	0.006
2006–2010	173,910	1356	0.008	0.007	0.008
2011–2015	108,313	747	0.007	0.006	0.007
Total	588,916	3747	0.006	0.006	0.007

**Table 9:** Third birth estimates for Kazakh and Russian women in Kazakhstan across five time periods, Kaplan–Meier estimates with confidence intervals

	Kazakh				Russian			
	Failure function	Std. error	[95%	conf.int.]	Failure function	Std. error	[95% Conf interval]	
*1989–1994*								
4 years since second birth	0.31	0.05	0.23	0.41	0.07	0.04	0.02	0.20
*1995–2000*								
4 years since second birth	0.28	0.01	0.26	0.31	0.10	0.02	0.06	0.15
6 years since second birth	0.40	0.02	0.37	0.43	0.14	0.03	0.09	0.21
10 years since second birth	0.54	0.03	0.49	0.59	0.17	0.04	0.11	0.25
*2001–2005*								
4 years since second birth	0.25	0.01	0.23	0.27	0.09	0.02	0.07	0.13
6 years since second birth	0.38	0.01	0.35	0.40	0.13	0.02	0.10	0.17
10 years since second birth	0.56	0.01	0.54	0.59	0.20	0.03	0.15	0.25
*2006–2010*								
4 years since second birth	0.35	0.01	0.32	0.37	0.10	0.02	0.07	0.14
6 years since second birth	0.55	0.01	0.52	0.57	0.14	0.02	0.11	0.18
10 years since second birth	0.72	0.01	0.70	0.75	0.29	0.03	0.23	0.36
*2011–2015*								
4 years since second birth	0.33	0.02	0.30	0.36	0.05	0.01	0.03	0.09
6 years since second birth	0.51	0.02	0.48	0.54	0.14	0.02	0.10	0.19
10 years since second birth	0.67	0.02	0.64	0.71	0.25	0.04	0.19	0.33

There are no women with spacing over 4 years contributing to the 1989–1994 synthetic cohort

**Table 10:** Exposure distributions for all variables for the study of fourth birth risks

	Person-month	Occurrences	Rate	[95% Conf interval]	
*Duration since the third birth*					
0–1 years since third child born	73,231	420	0.006	0.005	0.006
2–3 years since third child born	41,223	450	0.011	0.010	0.012
4–5 years since third child born	22,667	198	0.009	0.008	0.010
6–7 years since third child born	11,462	92	0.008	0.007	0.010
8–9 years since third child born	5006	38	0.008	0.006	0.010
10 and more years since third child born	1797	9	0.005	0.003	0.010
*Age at third birth*					
19 or less	165	3	0.018	0.006	0.056
20–24	16,529	180	0.011	0.009	0.013
25–29	72,141	655	0.009	0.008	0.010
30–34	48,836	309	0.006	0.006	0.007
35 +	17,715	60	0.003	0.003	0.004
*Ethnicity*					
Kazakh	129,092	1057	0.008	0.008	0.009
Russian	9695	35	0.004	0.003	0.005
Other	16,599	115	0.007	0.006	0.008
*Education*					
None/primary/not completed secondary	7765	58	0.007	0.006	0.010
Secondary	65,121	578	0.009	0.008	0.010
Secondary vocational	43,757	323	0.007	0.007	0.008
Higher	38,743	248	0.006	0.006	0.007
*Calendar period*					
1992–2000	18,527	133	0.007	0.006	0.009
2001–2005	48,248	355	0.007	0.007	0.008
2006–2010	49,872	423	0.008	0.008	0.009
2011–2015	38,739	296	0.008	0.007	0.009
Total	155,386	1207	0.008	0.007	0.008

**Table 11:** Fourth birth estimates for Kazakh and Russian women in Kazakhstan across four time periods, Kaplan–Meier estimates with confidence intervals

	Kazakh				Russian			
	Failure function	Std. error	[95%	conf.int.]	Failure function	Std. error	[95% Conf interval]	
*1992–2000*								
4 years since third birth	0.30	0.03	0.25	0.35	0.26	0.12	0.10	0.57
6 years since third birth	0.43	0.04	0.36	0.51				
*2001–2005*								
4 years since third birth	0.34	0.02	0.30	0.38	0.29	0.07	0.17	0.46
6 years since third birth	0.45	0.02	0.41	0.49	0.37	0.09	0.23	0.57
10 years since third birth	0.60	0.03	0.55	0.65				
*2006–2010*								
4 years since third birth	0.40	0.02	0.36	0.43	0.19	0.06	0.10	0.35
6 years since third birth	0.51	0.02	0.46	0.55	0.30	0.09	0.16	0.50
10 years since third birth	0.70	0.03	0.64	0.75	0.30	0.09	0.16	0.50
*2011–2015*								
4 years since third birth	0.33	0.02	0.29	0.37	0.07	0.04	0.02	0.20
6 years since third birth	0.49	0.02	0.44	0.53	0.10	0.05	0.04	0.25
10 years since third birth	0.66	0.04	0.59	0.73	0.20	0.10	0.07	0.50

There are no women with spacing over 4 years contributing to the 1992–2000 synthetic cohort among Russians and over 6 years among Kazakhs

## References

[CR1] Agadjanian V (1999). Post-Soviet demographic paradoxes: Ethnic differences in marriage and fertility in Kazakhstan. Sociological Forum.

[CR2] Agadjanian V, Dommaraju P, Glick J (2008). Reproduction in upheaval: Ethnic-specific fertility responses to societal turbulence in Kazakhstan. Population Studies.

[CR3] Agadjanian V, Dommaraju P, Nedoluzhko L (2013). Economic fortunes, ethnic divides, and marriage and fertility in Central Asia: Kazakhstan and Kyrgyzstan compared. Journal of Population Research.

[CR4] Aghajanian A (1991). Population Change in Iran, 1966–86: A stalled demographic transition?. Population and Development Review.

[CR5] Alam, A., & Banerji, A. (2000). *Uzbekistan and Kazakhstan: A tale of two transition paths.* Policy Research Working Paper; No. 2472. World Bank, Washington, DC. https://openknowledge.worldbank.org/handle/10986/19763

[CR6] Andersson G (2000). The impact of labour-force participation on childbearing behaviour: Pro-cyclical fertility in Sweden during the 1980s and the 1990s. European Journal of Population.

[CR60] Andersson G, Sobolev B (2013). Small Effects of Selective Migration and Selective Survival in Retrospective Studies of Fertility. European Journal of Population.

[CR7] Aydıngün A (2007). Islam as a symbolic element of national identity used by the nationalist ideology in the nation and state building process in post-Soviet Kazakhstan. Journal for the Study of Religions and Ideologies.

[CR8] Baizan P (2009). Regional child care availability and fertility decisions in Spain. Demographic Research.

[CR9] Becker G (1981). A treatise on the family.

[CR10] Billingsley S (2010). The post-communist fertility puzzle. Population Research Policy Review.

[CR11] Billingsley S (2011). Economic crisis and recovery: Changes in second birth rates within occupational classes and educational groups. Demographic Research.

[CR12] Blue L, Espenshade TJ (2011). Population momentum across the demographic transition. Population and Development Review.

[CR13] Burger O, DeLong JP (2016). What if fertility decline is not permanent? The need for an evolutionarily informed approach to understanding low fertility. Philosophical Transactions of the Royal Society B: Biological Sciences.

[CR14] Buyukkececi Z, Engelhardt H (2021). On the Relationship between fertility development and gender equality: A comparison of Western and MENA countries. Comparative Population Studies.

[CR16] Coale AJ (1984). The demographic transition. The Pakistan Development Review.

[CR17] Coleman D (2006). Immigration and ethnic change in low-fertility countries: A third demographic transition. Population and Development Review.

[CR18] Comolli CL, Neyer G, Andersson G (2021). Beyond the economic gaze: Childbearing during and after recessions in the Nordic Countries. European Journal of Population.

[CR53] Demoscope Weekly. (2019). *15 нoвыx нeзaвиcимыx гocyдapcтв. Кoэффициeнт cyммapнoй poждaeмocти. 1958–2018* [15 newly independent states. Total fertility rate, 1958–2018]. http://www.demoscope.ru/weekly/ssp/sng__tfr.php

[CR20] Frejka T, Gietel-Basten S (2016). Fertility and family policies in central and eastern Europe after 1990. Comparative Population Studies.

[CR21] Frejka T, Zakharov S (2013). The apparent failure of Russia’s pronatalist family policies. Population and Development Review.

[CR22] Friedman D, Hechter M, Kanazawa S (1994). A theory of the children. Demography.

[CR23] Fukai T (2017). Childcare availability and fertility: Evidence from municipalities in Japan. Journal of the Japanese and International Economies.

[CR24] Hakimian H (2006). From demographic transition to fertility boom and bust: Iran in the 1980s and 1990s. Development and Change.

[CR25] Hoem J, Kreyenfeld M (2006). Anticipatory analysis and its alternatives in life-course research: Part 1: Education and first childbearing. Demographic Research.

[CR15] Information-Analytical Centre. (2017). *National report on education system of the Republic Kazakhstan.* Ministry of Education and Science of the Republic of Kazakhstan. Retrieved from http://iac.kz

[CR26] Karaman Örsal DD, Goldstein JR (2018). The changing relationship between unemployment and total fertility. Population Studies.

[CR27] Kazimov R, Zakharov S, Goerres A, Vanhuysse P (2021). Combating low life expectancy and low fertility in tumultuous political times: A comparison of the Ukraine, Russia and Belarus. Global political demography.

[CR28] Kirk D (1996). Demographic transition theory. Population Studies.

[CR29] Kohler H-P, Kohler I (2002). Fertility decline in Russia in the early and mid-1990s: The role of economic uncertainty and labour market crises. European Journal of Population.

[CR30] Kreyenfeld, M. (2016). Economic uncertainty and fertility. In: Hank K., Kreyenfeld M. (eds) *Social Demography Forschung an der Schnittstelle von Soziologie und Demografie*. Kölner Zeitschrift für Soziologie und Sozialpsychologie. 10.1007/978-3-658-11490-9_4

[CR31] Legal Information System of Regulatory Legal Acts of the Republic of Kazakhstan (2022). Ministry of Justice of the Republic of Kazakhstan: Institute of legislation and legal information. https://adilet.zan.kz/eng

[CR32] Lesthaeghe, R., & Surkyn, J. (2004). When history moves on: The foundations and diffusion of a second demographic transition. Paper presented at the seminar on “Ideational perspectives on international family change”, Population Studies Center, Institute for Social Research (ISR), University of Michigan, Ann Arbor. http://sdt.psc.isr.umich.edu/pubs/online/WhenHistoryMovesOn_final.pdf

[CR33] Lesthaeghe R (2020). The second demographic transition, 1986–2020: Sub-replacement fertility and rising cohabitation: A global update. Genus.

[CR34] Notestein FW, Schultz TW (1945). Population-the long view. Food for the world.

[CR35] Oka, N. (2007). *Managing ethnicity under authoritarian rule: Transborder nationalisms in post-Soviet Kazakhstan*. Area Studies Centre, Institute of Developing Economies, JETRO.

[CR36] Omran AR, Roudi F (1993). The Middle East population puzzle. Population Bulletin.

[CR37] Perelli-Harris B (2005). The path to lowest-low fertility in Ukraine. Population Studies.

[CR38] Rakowska-Harmstone T (1977). Ethnicity in the Soviet Union. The Annals of the American Academy of Political and Social Science.

[CR39] Rindfuss RR, Guilkey DK, Morgan SP, Kravdal Ø (2010). Child-care availability and fertility in Norway. Population and Development Review.

[CR40] Sobotka T (2011). Fertility in central and eastern Europe after 1989: Collapse and gradual recovery. Historical Social Research/historische Sozialforschung.

[CR41] Sobotka T, Skirbekk V, Philipov D (2011). Economic recession and fertility in the developed world. Population and Development Review.

[CR42] Spoorenberg T (2013). Fertility changes in Central Asia since 1980. Asian Population Studies.

[CR43] Spoorenberg T (2014). Reconciling discrepancies between registration-based and survey-based estimates of fertility in Mongolia. Population Studies.

[CR44] Spoorenberg T (2015). Explaining recent fertility increase in Central Asia. Asian Population Studies.

[CR45] Spoorenberg T (2017). After fertility's nadir? Ethnic differentials in parity-specific behaviours in Kyrgyzstan. Journal of Biosocial Science.

[CR46] Statistics Agency, United Nations Children’s Fund. (2007). *Kazakhstan’s multiple indicator cluster survey 2006*. Final Report. Astana, Kazakhstan: Agency of Statistics, RK.

[CR47] Statistics Agency. (2010). *Итoги Haциoнaльнoй пepeпиcи нaceлeния 2009 гoдa в Pecпyбликe Кaзaxcтaн* [Results of the 2009 national population census in the Republic of Kazakhstan]. Astana, Kazakhstan: Agency of Statistics, RK.

[CR48] Statistics Agency, United Nations Children’s Fund. (2012). *Kazakhstan’s multiple indicator cluster survey 2010–2011*. Final report. Astana, Kazakhstan: Agency of Statistics, RK.

[CR49] Statistics Committee, United Nations Children’s Fund. (2016). *Kazakhstan multiple indicator cluster survey 2015*. Final report. Astana, Kazakhstan: The Statistics Committee of the MNE RK, UNICEF and UNFPA.

[CR51] Statistics Committee (2022). Кpaткиe итoги Haциoнaльнoй пepeпиcи нaceлeния 2021 гoдa в Pecпyбликe Кaзaxcтaн [Brief results of the 2021 National Population Census in the Republic of Kazakhstan].

[CR50] Statistics Committee. (2022b). *Dynamics of basic socio-economic indicators* [electronic resource]. Astana, Kazakhstan: Statistics Committee of the Republic of Kazakhstan. https://stat.gov.kz/

[CR52] Telebaev G (2003). *Peлигиoзнaя идeнтификaция нaceлeния и peлигиoзнaя cитyaция в Pecпyбликe Кaзaxcтaн* [Religious identification of population and religious situation in the Republic of Kazakhstan]. Coциoлoгичecкиe Иccлeдoвaния [Sociological Research].

[CR19] TransMonee Database. (2020). *Monitoring the situation of children and women in Europe and Central Asia. Kazakhstan country data.* https://transmonee.org

[CR54] Werner C, Emmelhainz C, Barcus H (2017). Privileged exclusion in post-soviet Kazakhstan: Ethnic return migration, citizenship, and the politics of (not) belonging. Europe-Asia Studies.

[CR55] Willmoth JR (2005). On the relationship between period and cohort mortality. Demographic Research.

[CR56] Wood J (2019). Social differentials in the effect of formal childcare on the transition to parenthood?. Advances in Life Course Research.

[CR57] Wood J, Neels K (2019). Local childcare availability and dual-earner fertility: Variation in childcare coverage and birth hazards over place and time. European Journal of Population.

[CR58] Yerekesheva LG (2020). Functions of religion and dynamics of nation-building in Kazakhstan and Uzbekistan. The Muslim World.

[CR59] Zakharov, S. (2008). Russian federation: From the first to second demographic transition. In: T. Frejka, T. Sobotka, J. Hoem, L. Toulemon (Eds), *Childbearing trends and policies in Europe*, Special Collection of *Demographic Research.**19*, 907–972. 10.4054/DemRes.2008.19.24

